# Nutrient‐driven regulation of saxitoxin gene expression and toxin production in *Raphidiopsis raciborskii* (Cyanobacteria)

**DOI:** 10.1111/jpy.70115

**Published:** 2025-12-03

**Authors:** Mehrzad Zare, Bruna Barçante, Juliana da Silva Martins Pimentel, Alessandra Giani

**Affiliations:** ^1^ Department of Botany, Phycology Laboratory Universidade Federal de Minas Gerais Belo Horizonte Minas Gerais Brazil; ^2^ Department of Genetic, Ecology and Evolution Universidade Federal de Minas Gerais Belo Horizonte Minas Gerais Brazil

**Keywords:** cyanotoxins, environmental stress, HPLC, Nostocales, RT‐qPCR, toxin analogs

## Abstract

*Raphidiopsis raciborskii* is a diazotrophic cyanobacterium, globally distributed in aquatic environments and known for forming toxic blooms, thereby affecting ecosystem services. South American strains are producers of saxitoxins, potent neurotoxins harmful to humans and animals. This study examined the effect of nutrient availability on saxitoxin production in two toxic *R. raciborskii* strains. Reverse transcription–quantitative polymerase chain reaction (RT‐qPCR) was used to investigate the transcriptional response of the saxitoxin *sxt*A4 gene under nitrogen and phosphorus gradients and the intracellular toxin concentration was measured by high performance liquid chromatography (HPLC). Results showed that the *sxt*A4 gene expression was generally upregulated at lower nutrient conditions. Positive correlations were observed among transcripts of *sxt*A4 and genes related to metabolic processes (*ntc*A, *nif*H, and *pst*S), an indication that nutrient stress may affect *sxt* gene regulation. Intracellular saxitoxin concentration increased slightly under moderate nitrogen reduction (10%), although not always significantly. Under phosphorus reduction, despite the observed upregulated transcription of *sxt*A4, total saxitoxin concentration significantly decreased, a possible consequence of hindered metabolic fitness. Interestingly, nutrient availability also affected the profiles of toxin analogs produced by *R*. *raciborskii*. Because different analogs exhibit variable toxicity, the presence of certain variants may enhance the toxic potential of an entire population under shifting environmental stressors. The responses observed in this study indicate the need for further investigations to identify the mechanisms controlling toxicity. This is particularly relevant as nutrient reduction may control cyanobacterial growth but not necessarily their toxin production.

**Abbreviations**
ANOVA: analysis of variance
CYN: cylindrospermopsin
dcGTXs: decarbamoyl‐gonyautoxins
dcneoSTX: decarbamoyl‐neosaxitoxin
dcSTX: decarbamoyl‐saxitoxin
doSTX: deoxy‐decarbamoyl saxitoxin
GTX: gonyautoxin
HPLC: high‐performance liquid chromatography
MAG: metagenome‐assembled genome
neoSTX: neosaxitoxin
*nifH*
: dinitrogenase reductase gene
NtcA: global nitrogen regulator
*ntc*A: global nitrogen regulator gene
PCR: polymerase chain reaction
PSP: paralytic shellfish poisoning
Pst: phosphate‐specific transport system
PstS: periplasmic phosphate‐binding protein
PSTs: paralytic shellfish toxins
qPCR: quantitative polymerase chain reaction
RT‐qPCR: reverse transcription–quantitative PCR
STX: saxitoxin
*sxt*
: STX gene cluster
SxtA: enzyme that initiate STX biosynthesis

## INTRODUCTION

*Raphidiopsis raciborskii* is a filamentous freshwater cyanobacterium with terminal heterocytes, belonging to the order Nostocales, family Nostocaceae (Aguilera et al., 2018; Gugger et al., 2005). This species has great ecological relevance due to the magnitude of its blooms, wide geographical distribution, and potential toxicity, since it can produce secondary metabolites such as the cyanotoxins cylindrospermopsin (CYN) and saxitoxin (STX), which can be extremely harmful to humans and animals (Bouvy et al., 1999; Carmichael et al., 2001; Pearson et al., 2010). The ability to produce either CYN or STX is related to the geographic origin of *R. raciborskii*. For instance, strains from Australia, Asia, and New Zealand typically produce CYN (Antunes et al., 2015; Rzymski & Poniedziałek, 2014; Wood & Stirling, 2003; Zare & Bahador, 2015), whereas South American strains produce STX (Hoff‐Risseti et al., 2013; Laux et al., 2023; Piccini et al., 2011).

Saxitoxin and its over 50 analogs form a large group of neurotoxic alkaloids, also known as the paralytic shellfish toxins (PSTs). Produced by dinoflagellates and some *Raphidiopsis raciborskii* strains, these toxins can accumulate in seafood, and human ingestion may cause paralytic shellfish poisoning (PSP), a severe and potentially fatal condition (Etheridge, 2010; Ibelings & Chorus, 2007; Landsberg et al., 2006; Lopes et al., 2017). The *sxt* gene cluster has been identified in different cyanobacterial genera, and although these clusters share a conserved core set of genes putatively responsible for STX biosynthesis, each organism may exhibit a different gene profile resulting in distinctive STX analogs (Wiese et al., 2010). In cyanobacteria, there are several specific enzymes, rare in microbial metabolism, that catalyze the biosynthesis of saxitoxins (Wiese et al., 2010). A chimeric origin has been suggested for the SxtA enzyme that is believed to be responsible for initiating STX biosynthesis. The process involves several reactions starting with acetate incorporation into the enzyme complex, followed by catalyzed methylation of the acetyl group and a Claisen condensation with arginine (Kellmann et al., 2008; Mihali et al., 2009; Moustafa et al., 2009; Wiese et al., 2010).

Saxitoxins are classified into three main groups based on structural variations: carbamoyl, decarbamoyl, and sulfocarbamoyl derivatives. The carbamoyl group comprises STX, neosaxitoxin (neoSTX), and gonyautoxins (GTX 1–4; Dittmann et al., 2013; Munday et al., 2013). The decarbamoyl analogs include decarbamoyl‐saxitoxins (dcSTX, dcneoSTX), decarbamoyl‐gonyautoxins (dcGTXs 1–4), and the 13‐deoxy‐decarbamoyl derivatives (doSTX, doGTX 2,3; Wiese et al., 2010). Several STX analogs have been reported in *Cylindrospermopsis raciborskii* (=*Raphidiopsis raciborskii*) T3, that is, saxitoxin, carbamoyl saxitoxin (STX, neoSTX, GTX2/3), decarbamoyl saxitoxin (dcSTX), and N‐sulfocarbamoyl (C1/2, B1) derivatives (Soto‐Liebe et al., 2010; Vico et al., 2016).

The ability of *Raphidiopsis raciborskii* to produce toxins and its increasing expansion from tropical to temperate regions (Antunes et al., 2015; Sinha et al., 2012) raises concerns in the context of water use and related public health matters. A key ecological advantage of this species is its aptitude for growth, and it can become very abundant or even dominant at varying nutrient levels, including at low concentrations of dissolved phosphorus (P) (Amaral et al., 2014; Bonilla et al., 2012; Willis et al., 2017) and dissolved nitrogen (N) (Dolman et al., 2012; Marques et al., 2022; McGregor & Fabbro, 2000; Vico et al., 2016).

Phosphorus is a major macronutrient essential for phytoplankton growth. Even though *Raphidiopsis raciborskii* has been reported mostly from eutrophic environments (Antunes et al., 2015; Batista et al., 2018; Burford et al., 2016), it has also been observed in low P environments thanks to its ability to rapidly take up and store P (Isvánovics et al., 2000; Posselt et al., 2009; Willis et al., 2017). *Raphidiopsis raciborskii* can also produce cytoplasmic polyphosphate granules, demonstrating high phenotypic plasticity and the ability to thrive under P‐limited conditions (Amaral et al., 2014; Rigamonti et al., 2018). Additionally, *R. raciborskii* can assimilate several forms of dissolved inorganic N (Spröber et al., 2003; Willis et al., 2016) and is capable of fixing atmospheric N_2_ as a diazotroph (Moisander et al., 2012; Sinha et al., 2014). As a result, the species has several strategies that enable its success even under poor N or P conditions, which makes predictions regarding the effect of nutrients on toxin production important to investigate.

Cyanobacterial saxitoxin production can be affected by several environmental factors that can also affect growth rate. Water temperature is a major environmental factor that influences cell growth and the production of STXs in PST‐producing Cyanobacteria (Castro et al., 2004; Cirés et al., 2017). Light intensity also affects the production of STXs and has been observed to promote STX production (Carneiro et al., 2009). Saxitoxin production has also been responsive to altered conditions of ionic strength and pH, indicating Na^+^ plays a role in the maintenance of cellular homeostasis (Ongley et al., 2016; Pomati et al., 2004).

The effect of nutrient availability on cyanobacteria toxin production has been tested in some previous studies (Rigamonti et al., 2018; Vico et al., 2016). For instance, in *Microcystis*, lower nutrient levels have been associated with increased production of the hepatotoxic microcystin (Alexova et al., 2016; Ginn et al., 2010; Pimentel & Giani, 2014), suggesting a potential function of microcystin in cellular stress response (Neilan et al., 2013). Some authors have also proposed that cyanotoxins may serve as evolutionary adaptations, providing competitive advantages and supporting enhanced physiological performance for their producers under adverse environmental conditions (Holland & Kinnear, 2013). Saxitoxin belongs to a different toxin class, but based on the aforementioned studies with microcystin, we aimed to investigate if N or P availability would influence the expression of genes involved in STX biosynthesis and STX production. This study intended to fill the current gap of knowledge linking gene expression to intracellular saxitoxin production in *Raphidiopsis raciborskii*. To follow the effects of N and P decreases on the species' physiology, known resource‐related genes were selected as markers and their expressions were related to *sxt* gene expression. For nitrogen, the global N regulator NtcA is known to activate the genes that are responsible for the assimilation and uptake of N (Herrero et al., 2001; Stucken et al., 2014), and for this reason, NtcA has been used as a marker in studies examining responses to N limitation (Lindell & Post, 2001; Pimentel & Giani, 2014). Additionally, the expression of the *nif*H gene, which encodes a subunit of the nitrogenase enzyme in N‐fixing cyanobacteria was evaluated. The presence of *nif*H transcripts under N limitation is an indication of the ability of *R. raciborskii* to use and fix atmospheric N_2_. Finally, for P, the P‐specific transport system (Pst) consisting of a periplasmic Pi‐binding protein (PstS), is upregulated in response to P deficiency (Wang et al., 2018); therefore, the expression of the *pst*S gene was used to assess the effects of P reduction.

The ability of *Raphidiopsis raciborskii* to grow across a wide range of nutrient concentrations indicates the importance of understanding whether nutrient availability can affect toxin production, which may help in predicting toxin presence in freshwater environments. A better knowledge of this response could be extremely valuable for managing cyanobacterial blooms and mitigating their impact on aquatic systems.

## MATERIALS AND METHODS

### Culture conditions and experimental design

Two toxic *Raphidiopsis raciborskii* strains (UFMG‐36 and UFMG‐186) were obtained from the Cyanobacteria and Algae Culture Collection of the Phycology Laboratory of Universidade Federal de Minas Gerais (Belo Horizonte, Brazil). Strains were originally isolated from Brazilian waterbodies (strain 36 from Lagoa Santa Lake and strain 186 from Pampulha Reservoir, both located in the state of Minas Gerais). Previous high performance liquid chromatography (HPLC) analyses in our laboratory (data not published) determined that both strains were able to produce saxitoxin and that strain 186 was slightly more toxic than strain 36; however, strain 36 produced a higher variety of toxins. Thus, both strains were selected to compare the response of strains with different toxin contents and profiles. Prior to the experiments, strains were maintained in WC medium (Guillard & Lorenzen, 1972), at a photoperiod of a 12:12 h light:dark cycle, an irradiance of ~ 40 μmol photons · m^−2^ · s^−1^, and a temperature at 22 ± 1°C.

For the experiments, different nutrient treatments were used by modifying nitrate (NaNO_3_) and phosphate (K_2_HPO_4_) concentrations in the standard WC medium; when decreasing the amount of NaNO_3_ and K_2_HPO_4_, these salts were replaced by sodium chloride (NaCl) and potassium chloride (KCl), to maintain ion balance with the equivalent amounts of Na and K, respectively. Strains were grown under the original NaNO_3_or K_2_HPO_4_ concentration in WC medium (control) and in two reduced conditions: medium and low N or P concentration (1/10 and 1/100 dilution, respectively, of NaNO_3_ and K_2_HPO_4_).

To ensure an acclimatization period, *Raphidiopsis raciborskii* strains were inoculated in the three different nutrient levels and grown under these conditions for ~ 1 week. After that, cultures were gently filtered through a cellulose membrane (0.8‐μm pore size, MFS‐Micro Filtration Systems) to remove all residual nutrients. The cyanobacterial cells retained on the filter were resuspended in 350 mL fresh medium at the three experimental nutrient concentrations and were allowed to grow for 2 days. Thereafter, subsamples were inoculated in triplicate in 250 mL at their respective concentrations, and the experiments were started. The experiments were finalized after 6 days. Several 20‐mL aliquots of each replicate were filtered through glass fiber filters (GF‐1, Macherey‐Nagel). Filters for chlorophyll and toxin measurements were kept frozen at −20°C until analysis. Filters for RNA extraction were immersed in TRIzol (Invitrogen) and kept at −80°C. Furthermore, 2 mL aliquots of each sample were harvested and fixed with Lugol solution (0.5%) for cell number quantification in a Fuchs Rosenthal hemocytometer. All treatments were performed in triplicate (three independent cultures for each treatment, under the same experimental conditions). All steps of the experimental procedure were performed under sterile conditions. Even though the experiments were performed in three replicates, some replicates were lost due to problems such as contamination; therefore, only two replicates of each treatment were used for the analyses in order to maintain balanced groups (equal numbers of replicates).

### RNA extraction and cDNA synthesis

For the RNA extraction, samples submerged in TRIzol (Invitrogen) were quickly deep‐frozen in liquid N and mechanically grounded by a homogenizer (Bio‐Rad ReadyPrep mini grinders adapted to a mini‐rotor). Afterward, the RNA was extracted according to TRIzol manufacturer's recommendations. Total RNA was suspended in 30 μL of Diethyl pyrocarbonate‐treated water (DEPC‐H_2_O), and RNA was treated with 0.5 μL of DNase (Promega) at 37°C for 30 min. In addition, 1.4 μL ethylenediaminetetraacetic acid (EDTA) 0.1 molar was added. The reaction was stopped by heating for 10 min at 65°C. Removal of DNA traces was verified by electrophoresis, and RNA was quantified using a Qubit RNA HS Assay (Thermo Fisher Scientific). The extracted RNA was subjected to reverse transcription (RT) to produce DNA copies (cDNA) with a HighCapacity kit (Applied Biosystems) with RT random primers. The polymerase chain reaction (PCR) cycling conditions were established according to the manufacturer's guidelines. The Applied Biosystems High‐Capacity cDNA Reverse Transcription Kit converts up to 2 μg of total RNA into single‐stranded cDNA using a 25°C (10 min) and 37°C (120 min) thermal cycle, relying on a system composed of 10× RT Buffer, 10× Random Primers, 25× dNTP Mix, and MultiScribe Reverse Transcriptase.

### Quantitative polymerase chain reaction (qPCR)

The obtained cDNA was used for RT‐qPCR to quantify the relative abundance of *sxt*A4, *ntc*A, *nif*H, and *pst*S transcripts. Real‐time PCR was applied using a StepOne system (Applied Biosystems) with 1 μL of cDNA of each sample, 0.3 μL of each primer (10 pmol · μL^−1^), 5 μL of Power SYBR Green I (Applied Biosystems), and sterile Milli‐Q water, for a final volume of 10 μL. The reactions were performed in four replicates. The specifications of the PCR cycle followed the manufacturer's guidelines. The PowerUp SYBR Green Master Mix is a 2× premixed solution containing the SYBR Green dye, Dual‐Lock Taq DNA Polymerase, dNTPs with dUTP/UDG for contamination control, and ROX reference dye. This mix is used for qPCR with a standard thermal cycle—including UDG activation at 50°C (2 min) and polymerase activation at 95°C (2 min), followed by 40 cycles of denaturation at 95°C (1–3 s) and annealing/extension at 60°C (30 s). All primer sets were amplified using this 60°C annealing temperature. We measured the transcript levels of genes involved in the STX gene (*sxt*A4), global N regulator gene (*ntc*A), N_2_‐fixation gene (*nif*H), and the gene coding for the PstS phosphate‐binding protein (*pst*S). Specific primers used in this study are described in Table 1. To amplify 132‐base pair products for the *pst*S gene, a new primer was designed with the help of Primer‐BLAST tools (National Center for Biotechnology Information). As in previous studies, the primer described for the 16S rRNA gene was used as a housekeeping gene (Kaebernick et al., 2000; Marques et al., 2022; Pimentel & Giani, 2014; Wu et al., 2013). Relative quantification of the *sxt*A4, *nif*H, *ntc*A, and *pst*S target genes was calculated with the 16S rRNA gene as a reference gene. We followed Pfaffl (2001) and ran correlation analyses of the threshold cycle values where the Δ*C*_T_ = *C*_T_ target gene – *C*_T_ housekeeping gene among *sxt*A4, *nif*H, *ntc*A, and *pst*S. Changes in expression between treatments were calculated according to the formula Log_2_(2^−ΔΔ*C*t^), where ΔΔ*C*t = Δ*C*_T_ treatment – Δ*C*_T_ control.

**TABLE 1:** Amplification primers used in this study.

Primer	Type	Sequence 5′–3′	Amplicon size (bp)	Function	References
sxtA4 sxtA4	F R	GGACTCGGCTTGTTGCTTC CCAGACAGCACGCTTCATAA	200	*sxt*A gene (Initiatior of STX biosynthesis)	Hoff‐Risseti et al. (2013)
ntcA ntcA	F R	TGCGGTGGAATTGCTCTCTT CTGTTTGCAGAATCCGCGAG	113	Global nitrogen regulator	Marques et al. (2022)
nifH nifH	F R	CGTAGGTTGCGACCCTAAGGCTGA GCATACATCGCCATCATTTCACC	297	N_2_ fixation	Gugger et al. (2005)
pstS pstS	F R	AAGCTGGGACGGTATTTGGGGG TACCAAACGTCCTATGCGCC	132	Coding for phosphate‐binding periplasmic protein	This study
16S‐rRNA 16S‐rRNA	F R	AGAAAAGAGGTTTACGACCCAAGAGC TGAAAGATTTATTGCCTGGAGATGAGC	267	Housekeeping gene	Wu et al. (2013)

Abbreviations: F, forward; R, reverse.

The amplification melting temperature of the PCR products was identified by analyzing the fluorescent melting curve under a steadily rising temperature at a 0.1°C · s^−1^ rate from 70 to 95°C. The fluorescence intensity was continuously measured and finally converted to melting peaks with the LightCycler software (StepOne Software, version 2.0). The melt curve analysis was performed after every qPCR run for each primer, and only reactions that produced a single, sharp, and specific peak were considered valid. Polymerase chain reaction efficiency for each primer pair was determined using standard curves generated from serial dilutions of pooled cDNA. All primer sets exhibited efficiencies between 90% and 110%, with correlation coefficients (*R* ^2^) above 0.98.

### Intracellular toxin measurements

The analyses of the STX carbamoyl group including STX, neoSTX, GTX 1–4, and dcSTX were performed by HPLC according to Oshima (1995) and Van de Riet et al. (2011). Saxitoxins were extracted with a 0.05 M acetic acid solution, through sonication of the samples and successive centrifugation at 10,000 rpm (three cycles). Supernatants were recovered and filtered (0.45 μm, mod. PTFE, Millipore) into vials. Chromatographic analyses were performed with a Waters Alliance 2695 HPLC system coupled with a fluorescence detector (Waters 2475) and equipped with a reversed phase C8 silica‐based column (Waters Symmetry). All solvents were liquid chromatography (LC) grade or analytical grade for the post‐column derivation. The chromatographic procedure consisted of elution under isocratic conditions using the following mobile phase: 2 mM sodium heptane‐sulfonate in 30 mM phosphoric acid (pH 7.1) containing 4.8% (v/v) of acetonitrile (flow rate 0.8 mL · min^−1^). Post‐column derivatization was carried out in an oven at 85°C (Waters Post‐column reaction module), with the oxidizing reagent prepared from 7 mM periodic acid and 50 mM dipotassium phosphate (pH 9.0) and the acidifying reagent of 0.5 M acetic acid (flow rate 0.4 mL · min^−1^). The identification and quantification of compounds were performed using the fluorescence detector with excitation at 330 nm and emission at 390 nm. Peaks were identified and quantified by comparison with the retention time and integrated area of the standards. Calibration curves were prepared for each analog using serial dilutions and correlation coefficients were *R* ^2^ > 0.99. Certified analytical standards of STXs were obtained from CIFGA Laboratory (Spain).

### Chlorophyll *a* measurement

Chlorophyll *a* concentration was measured according to Nusch (1980). Chlorophyll *a* was extracted from the frozen filters in hot ethanol and measured in a spectrophotometer (PerkinElmer) at a 665‐nm wavelength.

### Statistical analyses

Statistical analyses were performed by using SPSS software (version 26). After a two‐tailed Levene's test for equality of variances, one‐way analysis of variance (ANOVA) was applied to analyze differences among means of the relative gene expression and intracellular toxins in the treatments under different nutrient concentrations. *F* critical values were used to retain or reject the null hypothesis; when the *F*‐statistic obtained by ANOVA was greater than the critical *F*‐value, the null hypothesis was rejected, confirming significant difference. A post hoc Tukey honestly significant difference test was applied to verify differences among the means of all group pairs. Significant differences were accepted for *p* ≤ 0.05. Correlation analyses were performed between Δ*C*_T_ of *sxt*A4 and *ntc*A, *nif*H, and *pst*S merging together results from both strains and experimental repetitions at different nutrient concentration.

## RESULTS

### Gene response under nitrogen and phosphorus gradient

To quantify the gene expression of the reduced nutrient treatments, that is, at medium (10% N or P) and low (1% N or P) concentrations, the treatment with the highest nutrient concentration (control) was always used as a reference and baseline condition.

**FIGURE 1:**
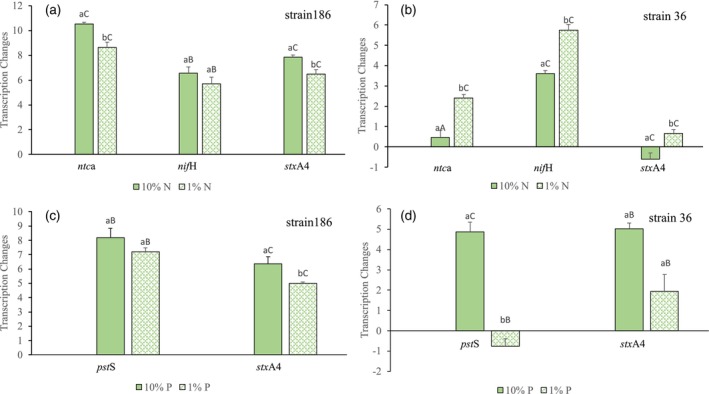
Fold change (log_2_ relative quantification) of *ntc*A, *nif*H, *pst*S, and *sxt*A4 gene expression in the nitrogen (N) and phosphorus (P) assays for two *Raphidiopsis* strains (a, c: UFMG‐186 and b, d: UFMG‐36). The original N and P concentration (100%) was used as control and baseline conditions (indicated by 0 on the *y‐*axis) against which treatments were compared to estimate changes in gene expression under decreased concentration: Medium (10%) and low (1%). Lowercase letters above the bars indicate significant differences for each gene between the two treatments, and uppercase letters indicate significant differences when treatments were compared to the control. Whiskers indicate standard errors.

In the nitrogen experiment, the analysis of the *ntc*A, *nif*H, and *sxt*A4 transcription changes showed that the genes were generally upregulated under reduced N concentrations (Figure 1a,b). However, the regulatory patterns differed between strains in the nitrogen levels that maximized gene expression. For strain 186, the *ntc*A gene was upregulated in both N concentrations (*F*_2,3_ = 125.89, *p* < 0.001), whereas in strain 36, a significant increase in the expression level was only observed under the lowest N concentration (*F*_2,3_ = 37,90, *p* < 0.01; Figure 1b). In both strains (186 and 36), a significant increase in *nif*H gene expression was also recorded in both nitrogen conditions as compared to the control (strain 186: *F*_2,3_ = 77.85, *p* < 0.01; strain 36: *F*_2,3_ = 130.29, *p* < 0.001). The *sxt*A4 gene expression showed changes under both reduced nitrogen conditions. In strain 186, it had a significant increase (*F*_2,3_ = 335.07, *p* < 0.001) under both concentrations as compared to the control (Figure 1a), whereas in strain 36, a significant increase was only observed at 1% N concentration, that is, when nitrogen was reduced 100 times compared to the control condition (*F*_2,3_ = 101.28, *p* < 0.002; Figure 1b).

In the phosphorus experiment, the *pst*S gene was upregulated in strain 186 in both P concentrations compared to the control (*F*_2,3_ = 66.28, *p* < 0.01; Figure 1c). However, in strain 36, the expression of the *pst*S gene (Figure 1d) only increased under the medium P concentration and was down‐regulated at 1% P (*F*_2,3_ = 130.29, *p* < 0.001). Both strains showed increases in *sxt*A4 gene expression under reduced P conditions (Figure 1c,d), with a significantly higher expression of the *sxt*A4 gene in the medium P concentration (10% P), when phosphorus was reduced 10 times compared to the control (strain 186: *F*_2,3_ = 335.07, *p* < 0.001; strain 36: *F*_2,3_ = 12.23, *p* < 0.05). At 1% P, the difference was less, but the *sxt*A4 gene was still upregulated and significantly higher than the control.

As a whole and as expected, the *ntc*A, *nif*H, and *pst*S genes showed increased expression under nitrogen and phosphorus reduction. The analysis of *sxt*A4 gene expression also recorded general trends of upregulation under these experimental nutrient conditions, suggesting that nutrient stress could trigger toxin production.

**FIGURE 2:**
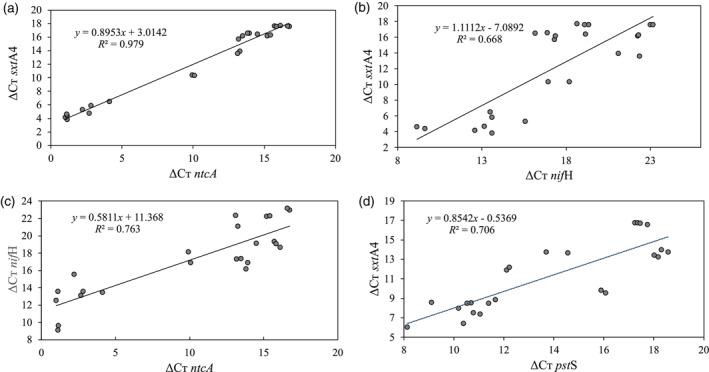
Correlation analyses between Δ*C*_T_ values of the expression of (a) *ntc*A and *sxt*A4, (b) *nif*H and *sxt*A4, (c) *ntc*A and *nif*H genes for all experimental data obtained from the nitrogen experiments (*n* = 24), and (d) *pst*S and *sxt*A4 genes for all experimental data obtained from the phosphorus experiments (*n* = 24).

Pairwise comparisons—*ntc*A and *sxt*A4, *nif*H and *sx*tA4, and *ntc*A and *nif*H (Figure 2a–c)—for the N experiments, as well as between *sxt*A4 and *pst*S for the P experiments, (Figure 2d) all had positive correlations between all pairs of genes (*R* ^2^ values were always close to or above 0.7). The highest correlation coefficient was observed between *ntc*A and *sxt*A4 (*R* ^2^ = 0.979; Figure 2a).

### Intracellular toxins and chlorophyll *a*

Intracellular toxins and chlorophyll *a* have been presented on a per‐cell basis and were calculated based on quantification of cell numbers in each experimental vessel (Figure S1).

The analyses performed by HPLC on the two strains detected the presence of toxins belonging to the carbamoyl group (STXs) and included STX, NeoSTX, GTX 1–4, and dcSTX. Representative chromatograms have been included as Figures S2–S6.

**FIGURE 3:**
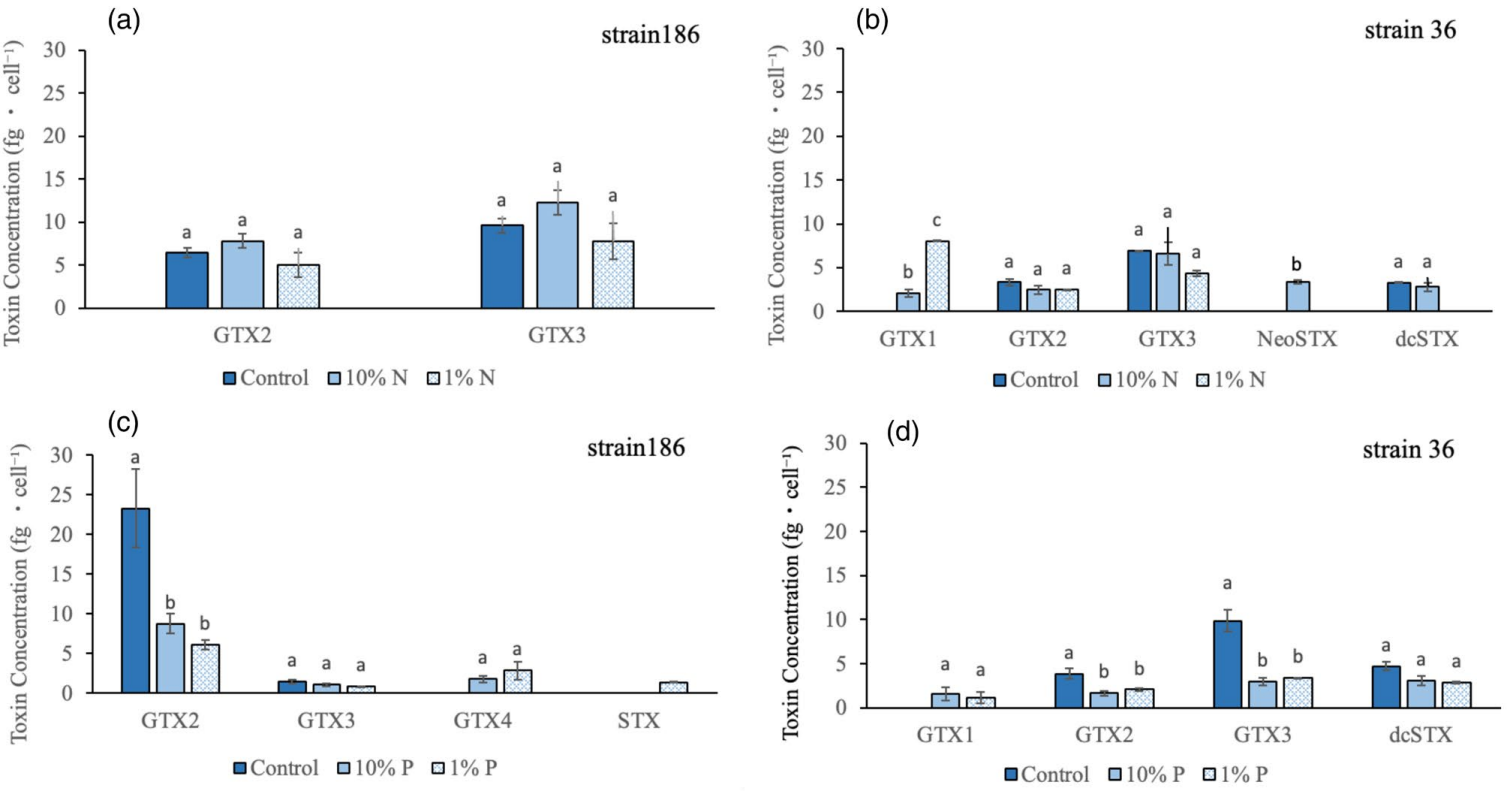
Toxin concentrations per cell for strains UFMG‐186 (a, c) and UFMG‐36 (b, d) at three nitrogen (N) and phosphorus (P) concentrations: Original N and P concentration (control: 100%), medium (10%), and low (1%). Different letters above the bars indicate significant differences (*p* ≤ 0.05) among treatments. Values represent averages. Whiskers indicate standard errors.

In the nitrogen experiment, strain 186 showed the presence of only two gonyautoxin variants (GTX2 and GTX3; Figure 3a), whereas five different toxins were identified in strain 36 (GTX1, GTX2, GTX3, NeoSTX, and dcSTX; Figure 3b). Although the amounts of GTX2 and GTX3 in strain 186 were higher in the medium N concentrations (10% N), statistical analysis showed that differences between the control and the two N conditions were not significant (GTX2: *F*_2,3_ = 2.01, *p* > 0.05; GTX3: *F*_2,3_ = 2.16, *p* > 0.05). In strain 36, however, we observed that the toxin profile changed according to the N level. For example, GTX1 was not detected in the control but was significantly increased in both reduced N concentrations (*F*_2,3_ = 337.80, *p* < 0.001), especially at 1% N. Neosaxitoxin was only identified at 10% N (*F*_2,3_ = 250.90, *p* < 0.001) and production of dcSTX was suppressed at 1% N (*F*_2,3_ = 37.36, *p* < 0.01). Approximately the same amounts of GTX2 and GTX3 toxins were produced in all N treatments with no significant differences among them in strain 36 (GTX2: *F*_2,3_ = 1.912, *p* < 0.05; GTX3: *F*_2,3_ = 3.320, *p* > 0.05).

In the phosphorus experiment, GTX2, GTX3, GTX4, and STX were identified in strain 186 (Figure 3c); GTX2 significantly decreased in both P‐reduced treatments compared to the control (*F*_2,3_ = 339.56, *p* < 0.05). GTX3 was produced in all P treatments, with no significant difference among treatments (*F*_2,3_ = 3.31, *p* > 0.05). GTX4 was only detected after P reduction at 10% and 1%, with no significant difference between these two P levels. Saxitoxin was produced only at the 1% P treatment (*F*_2,3_ = 571.68, *p* < 0.001). In strain 36, GTX1, GTX2, GTX3, and dcSTX toxins were detected (Figure 3d). GTX1 was only produced in the treatments after P reduction, with no significant differences among the treatments (*F*_2,3_ = 2.18, *p* > 0.05). The production of GTX2 and GTX3, however, decreased significantly under P reduction as compared with the control (*F*_2,3_ = 26.69, *p* < 0.01). Lastly, dcSTX did not show significant difference among P treatments (*F*_2,3_ = 5.90, *p* > 0.05).

**FIGURE 4:**
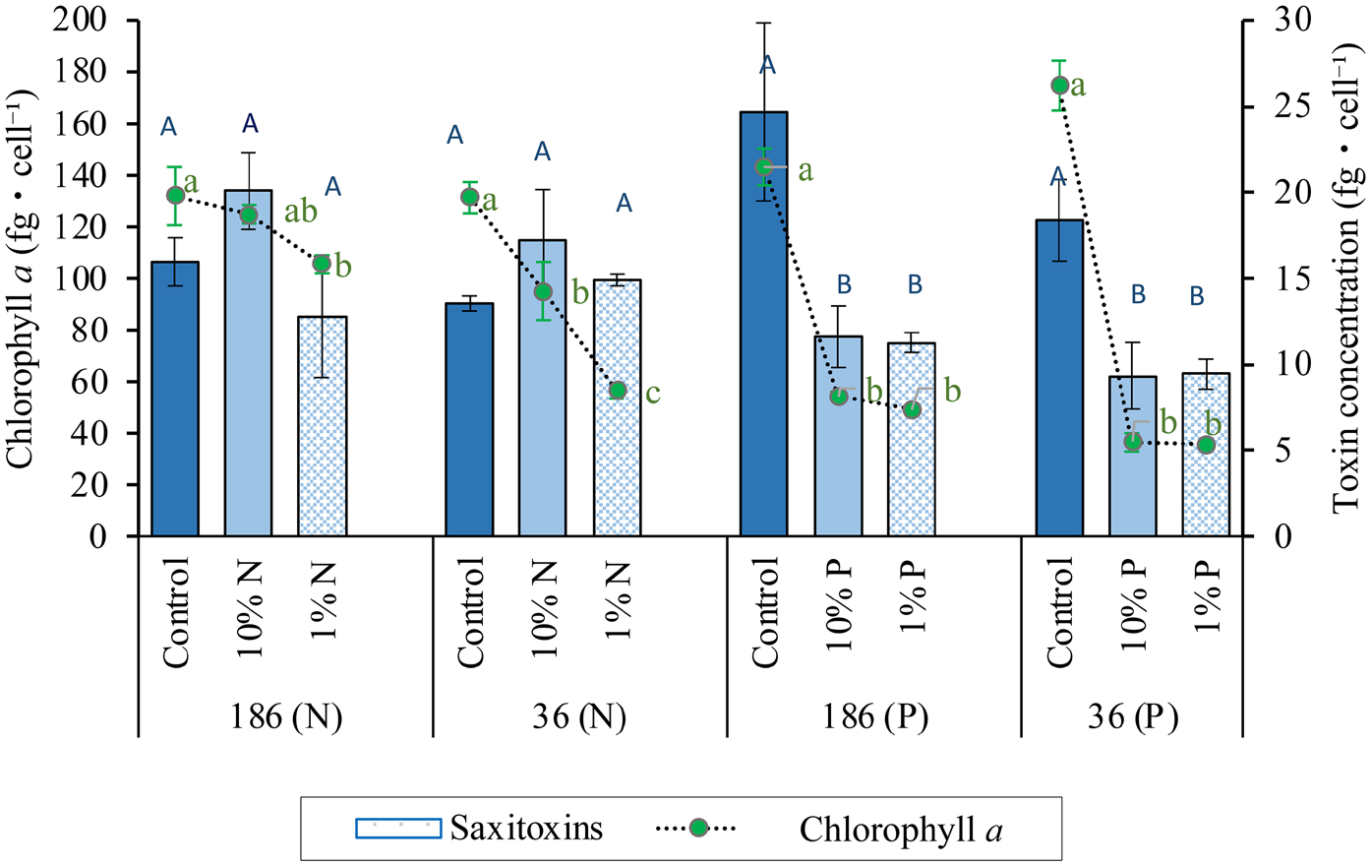
Chlorophyll *a* and total toxins content per cell for each strain (UFMG‐186 and UFMG‐36) at three nutrient concentrations in the nitrogen (N) and phosphorus (P) experiments. Different lowercase letters beside the green circles indicate significant differences in chlorophyll concentration (*p* ≤ 0.05); different capital letters above bar charts indicate significant differences in total toxins concentration (*p* ≤ 0.05). Values represent averages and were compared to the control and among themselves for each strain. Whiskers indicate standard errors.

In summary, even when significant differences were observed in the composition of individual toxins among N treatments, the amount of total STX showed a trend of higher toxin content at 10% N, but that trend was not significant (strain 186: *F*_2,3_ = 2.098, *p* > 0.05; strain 36: *F*_2,3_ = 1.23, *p* > 0.05; Figure 4). In contrast, a significant decrease in total toxin content was observed in the P experiments at the two reduced P levels for both strains (strain 186: *F*_2,3_ = 13.15, *p* < 0.05; strain 36: *F*_2,3_ = 13.16, *p* < 0.05).

As expected in photosynthetic autotrophs growing under nutrient reduction, cellular chlorophyll *a* concentrations decreased, albeit at different degrees, as the amount of N and P provided to the culture medium was reduced, confirming that the experimental conditions caused metabolic changes in the cells (Figure 4). In the N experiment, strain 186 showed a significant difference in chlorophyll *a* concentration only between the control and 1% N concentration (*F*_2,3_ = 9.036, *p* = 0.05), whereas in strain 36, there were significant differences among all treatments (*F*_2,3_ = 675.39, *p* < 0.001). In the P experiment, chlorophyll *a* in both strains showed significant and similar decreases in both P concentrations as compared to the control (strain 186 *F*_2,3_ = 56.39, *p* < 0.005, *p* = 0.001; strain 36 *F*_2,3_ = 249.75, *p* < 0.001). There were no significant differences between the two P reduced concentrations for either strain. These results show that the significant upregulation of *sxt*A4 did not consistently lead to increased intracellular toxin concentration, especially under reduced P conditions.

## DISCUSSION

Based on the previous studies described in the introduction that reported the potential influence of environmental factors and stress conditions on toxin production in *Raphidiopsis raciborskii*, we tested the hypothesis that the expression of genes involved in saxitoxin synthesis would be upregulated when *R. raciborskii* strains were exposed to nutrient reduction, specifically to N and P reduction. In agreement with this hypothesis, our results showed that the expression of the *sxt*A4 gene increased in strains growing under reduced concentrations of N and P, demonstrating that *sxt*A4 transcription responds to nutrient stress. This result was reinforced by the positive and significant correlations observed between the *ntc*A, *nif*H, and *pst*S genes, which are related to nutrient metabolism, and *sxt*A4 gene transcription.

NtcA, an activator regulatory protein, is a known and reliable marker for investigating N limitation, since it activates N‐responsive genes (Lindell & Post, 2001). In cyanobacteria, N control is performed by NtcA at the transcriptional level (Álvarez‐Escribano et al., 2018; Herrero et al., 2001). This protein is required for gene expression in the ammonium and nitrate uptake pathways and for heterocyte development when present (Wei et al., 1993, 1994). Under N deprivation, the *ntc*A gene is quickly induced and autoregulated (Muro‐Pastor et al., 2002; Ramasubramanian et al., 1996; Wei et al., 1994). Lindell and Post (2001) reported that *ntc*A gene transcription in *Synechococcus* sp. strain WH7803 was enhanced when ammonium levels declined to 1 mM, whereas the increase of more than 1 mM ammonium caused a quick decrease in *ntc*A gene transcription. Furthermore, using the same strain, the authors reported the highest level of *ntc*A gene transcription under conditions of N deprivation. Thus, higher transcription of *ntc*A generally results from N shortage (Lindell & Post, 2001).

The common model of transcription anticipated for NtcA involves an enhancement in transcription. Afterward, while the manufacturing of the activator protein increases, the transcription of the target protein is upregulated as well (Ginn et al., 2010). Ginn et al. (2010) reported that in *Microcystis aeruginosa* PCC 7806 under N limitation, the expression of *mcy*B, a gene related to microcystin production, was matched by the enhancement of *ntc*A expression, suggesting that microcystin production can be influenced by N levels. Pimentel and Giani (2014) also observed that nitrogen stress, either in the form of nitrate or ammonium, induced *ntc*A and *mcy*D expression in *M. aeruginosa* strains, with a significant correlation between the two genes. Some authors have suggested that the correlation between the *ntc*A gene and the *mcy* gene region may be an effective indication of an intracellular function for microcystin (Ginn et al., 2010; Muro‐Pastor et al., 2002). These patterns of *ntc*A and *mcy* expression are consistent with the patterns of *ntc*A and *sxt*A4 expression observed here.

The *nif*H gene encodes the dinitrogenase reductase component of the nitrogenase enzyme complex and is conventionally used as a marker for the process of N_2_ fixation (Latysheva et al., 2012; Marques et al., 2022; Sinha et al., 2014). In cyanobacteria of the Nostocales order, the heterocytes are the only sites for *nif*H transcription and NifH biosynthesis (Elhai & Wolk, 1990; Plominsky et al., 2013). Marques et al. (2022) observed that in *Raphidiopsis raciborskii* strains, the transcription of *nif*H, *het*R (a regulatory gene involved in heterocyte differentiation), and *ntc*A genes, as well as the number of heterocytes, increased under N limitation, consistent with the results of our study. Therefore, in N‐limited conditions, *R. raciborskii* cells may be able to obtain N from biological fixation.

The Pst operon consists, in part, of a periplasmic Pi‐binding protein (PstS) that sensitively responds to P deficiency (Wang et al., 2018). For this reason, the expression of the *pst*S gene has been used to assess P limitation. Wang et al. (2018) reported that in *Dolichospermum flos‐aquae*, a diazotrophic cyanobacterium, the transcription of the *pst*S gene in treatment without P was significantly upregulated and 2.8 times higher in comparison with high P‐level conditions. Scanlan et al. (1997) also showed that in *Synechococcus* sp. strain WH 7803, PstS was increased under lower ${\mathrm{PO}}_{4}^{-}$ concentrations. Our results of increased *pstS* expression in reduced 10% P are in agreement with these findings in the literature.

Kellmann et al. (2008) first described a putative STX biosynthetic gene cluster (*sxt*) encoded by more than 35 kb in *Cylindrospermopsis raciborskii* T3, with 30 catalytic functions to 26 proteins. These authors observed two transcriptional factors related to PhoU and OmpR, respectively: *sxt*Y and *sxt*Z. The phosphate (Pho) regulon plays a key role in phosphate homeostasis and part of it is encoded together with proteins of the phosphate‐specific transport (Pst) system; proteins related to PhoU are negative regulators of phosphate uptake, whereas proteins like OmpR may be part of the regulation of several metabolic processes, such as those, for example, related to N metabolism and osmoregulation (Kellmann et al., 2008; Pearson et al., 2010). Interestingly, Mihali et al. (2009) did not find the *sxt*Y, *sxt*Z, or *omp*R gene to be involved in signal transduction and transcriptional regulation, in the gene clusters characterized for *Aphanizomenon* and *Anabaena*, suggesting either those genes might be regulated differently or the genes may have been transposed to different loci in the genome. In a study on *Raphidiopsis raciborskii* metagenome‐assembled genomes (MAGs) from the Pampulha Reservoir (Brazil), Laux et al. (2023) observed that *sxt*Z and *sxt*Y were positioned closer to the Pho regulon, namely the P regulon sensor protein PhoR (*sxt*Z) and the Pst regulatory protein PhoU (*sxt*Y). In their study however, the complete saxitoxin cluster was not observed in the same genomic region, but it can be challenging to determine genomic arrangements with MAG. Kellmann et al. (2008) and Pearson et al. (2010) suggested that STX production in *Cylindrospermopsis raciborskii* T3 could be regulated at the transcriptional level in response to P availability and other environmental factors. As previously mentioned, we observed significant upregulation of the *sxt*A4 gene in the N‐ and P‐reduced conditions relative to the control, even though some differences were observed between the two strains in their gene transcription changes.

Rigamonti et al. (2018), studying *Raphidiopsis* strains that produced cylindrospermopsin, observed a significant twofold increase in *cyr*A gene expression in N‐deprived conditions (−N + P) as compared to the control (+N + P). They also reported that the total CYN concentration was considerably higher in the −N + P treatment when compared to the control (Rigamonti et al., 2018). Under P deficiency, however, the relative expression of the *cyr*A gene was down‐regulated (Rigamonti et al., 2018). In a similar investigation, Saker and Neilan (2001) observed that *Cylindrospermopsis raciborskii* cultures grown without an N source had the highest CYN concentrations, but they did not evaluate gene expression. Yang et al. (2018) reported higher CYN production at lower N levels but, also, without presenting any measurements of gene expression. Stucken et al. (2014) observed that in both CYN‐ and STX‐producing species the NtcA binding boxes were located within the gene clusters suggesting a potential role of NtcA as a regulator of toxin biosynthesis. However, Stucken et al. (2014), did not identify an effect of N on *cyr* or *sxt* expression. Investigating the effect of nutrients on CYN production, Bácsi et al. (2006) noted that starvation for phosphorus and sulfate caused a reduction of the cellular CYN content in *Aphanizomenon ovalisporum*, whereas N starvation did not influence CYN content. In sum, N has variable impacts on toxin gene expression and toxin concentrations reinforcing the importance of evaluating responses systematically across strains and species.

In a study concerning an STX‐producing *Aphanizomenon* strain, Dias et al. (2002) observed that changes in nutrient (N and P) levels affected toxicity. They detected higher toxin quotas under nitrate depletion and suggested that the potentially detrimental effects of N limitation on the size and composition of the free intracellular amino acids could be partially lowered in heterocytous N‐fixing cyanobacteria. This could also be the case of *Raphidiopsis raciborskii* in our study, since *nif*H gene expression was significantly upregulated under reduced N and could have diminished the effect of N reduction in our short‐term experiments.

In our study, despite the significant upregulation of the *sxt*A gene observed in both strains under N and P reduction and the positive correlations observed between STX gene expression and cellular metabolic changes expressed by the *ntc*A, *nif*H, and *pst*S genes, the amount of total intracellular toxins did not increase in the reduced N or P treatments. There was no change in toxin content increase in the reduced N experiment, and the toxin concentration significantly decreased by around half in the reduced P experiment. The difference observed between the two nutrients seems to indicate that *Raphidiopsis raciborskii* is more resistant to low N than to low P, possibly related to its ability to produce heterocytes and fix nitrogen (Dias et al., 2002; Marques et al., 2022; Sinha et al., 2014). In contrast, it seems that the lack of intracellular P, even though it promoted an upregulation of the *sxt* gene, hindered the production of toxins. Because several enzymes involved in the toxin production are ATP‐dependent, those enzymes could have been inhibited under P reduction (Dias et al., 2002; Mazard et al., 2012). The reduction of STX production in low P, but not low N, was reinforced by the results recorded in our experiments for chlorophyll *a* content per cell, which were significantly and more sharply reduced at lower P concentrations when compared to N. Again, this may be a function of the ability to augment N demand with fixed N. These physiological responses indicated that *R. raciborskii* may be able to save resources by modifying and adapting metabolic pathways under reduced nutrient conditions, a response previously observed in the potentially toxic cyanobacterium *Microcystis aeruginosa* (Ginn et al., 2010; Pimentel & Giani, 2014; Steffen et al., 2014).

Despite the limited changes in the toxin content in our experiments, we observed that the toxin profile of our strains differed under different culture conditions, with a total of four analogs being recorded for strain 186 and five for strain 36. Additionally, certain variants were observed only under nutrient reductions: GTX1 and NeoSTX in strain 36 (10% and 1% N) and GTX4 and STX in strain 186 (10% and 1% P). As previously documented by several authors, different isolates of cyanobacterial species may be toxic or not and may have several toxin profiles (Ballot et al., 2010; Miotto et al., 2017; Murray et al., 2011). The toxin composition seems to be controlled by the position and presence or absence of the genes in the cluster (Boopathi & Ki, 2014) that define the toxic phenotype. Saxitoxin analogs are derivatives of the parent STX molecule (Mihali et al., 2009) as a direct result of the evolution of the genes present in the genome of the organism and of the tailoring enzymes encoded by the *sxt* gene cluster (Wiese et al., 2010). To assess the “in vivo” activity and specificity, Soeriyadi et al. (2021) heterologously expressed SxtA and observed that the enzyme had a broad specificity capable of condensing various substrates; thus, it is expected that reactions catalyzed by SxtA could produce multiple STX analogs.

Changes in the toxic profiles in cultures submitted to different growth conditions were also observed in previous studies. Dias et al. (2002) suggested that changes in the relative proportion of STX analogs in *Aphanizomenon* LMECYA growing under P limitation could be ATP dependent, and therefore, production of certain variants could be inhibited by the lack of P. The authors observed an increase in the proportion of STX and dcSTX under P limitation and an increase of GTX5 and NeoSTX in P‐rich medium. Although they observed a shift in the toxin profile, the pattern of analog changes differed from our results, possibly due to the different cyanobacterial species and nutrient levels used in their experiments (Dias et al., 2002). Rangel et al. (2016) also noticed that different temperatures could result in changes in the STX composition of *Raphidiopsis raciborskii* strains and that the concentration of certain analogs increased at higher temperatures. For instance, at higher temperatures, NeoSTX tended to increase, and the dcSTX analog was only detected at higher temperatures (Rangel et al., 2016), so temperature variation as well as strain heterogeneity could underpin variability in toxin profiles.

Additionally, it is important to remark that different STX analogs have different toxicities (Ballot et al., 2010; Dias et al., 2002; Munday et al., 2013; Wiese et al., 2010). Therefore, environmental changes may affect the toxic biomass not only by changing the total toxin production but also by altering the toxin profiles of the organisms and, in consequence, increasing the potential toxicity of a bloom. In a comprehensive study on the relative toxicity of STX and its derivatives, Munday et al. (2013) observed that NeoSTX and GTX‐1 and GTX‐4 were significantly more toxic than STX (by factors of 3.2 for NeoSTX and 1.9 for GTX‐1 and GTX‐4), emphasizing the importance of the relative components in a mixture. Furthermore, some authors have stated that because of the partial differences in their chemical properties, different analogs could potentially have differing intracellular functions (Wiese et al., 2010), implying that some changes in the toxin profile could be advantageous in particular environmental conditions as additional adaptations to stress.

The current evidence regarding the role of N and P on STX production by *Raphidiopsis raciborskii* seems to be contradictory in some studies, even if many studies have indicated an increase in toxin production or gene expression under nutrient reduction. Factors such as nutrient concentrations, incubation time, and target genes, among others, may all affect the results and may, in part, explain these differences. The role of saxitoxins in *R. raciborskii* remains to be elucidated, but the fact that under lower nutrient availability STX is still produced and that its toxin profile can be altered by changes in physiology, may point to its potential role as a stress regulator in cell metabolism. Experiments running over a longer time interval could help verify if upregulated gene expression translates into higher toxin production in the cell, since in our short‐term experiments the gene‐expression response may have been faster than the metabolic process implicated in the production of the compound, in this case STX. Therefore, the short experimental duration could have potentially masked delayed metabolic responses.

It has been suggested that shifts in enzymatic activity or posttranscriptional mechanisms are involved in toxin biosynthesis and regulation (Stucken et al., 2014), potentially explaining the delayed response in toxin production observed here. Late regulatory responses were observed by Stucken et al. (2014) in an experiment measuring toxin production in *Cylindrospermopsis raciborskii* and *Raphidiopsis brookii*. Posttranscriptional mechanisms are critical in regulating gene expression, allowing cells to rapidly adapt to environmental changes and stressors and adjust protein production according to cellular developmental needs (Martinez & Vadyvaloo, 2014). In a recent study on the biosynthesis gene cluster of the toxin cylindrospermopsin in *R. raciborskii*, Cullen et al. (2025) proposed additional posttranscriptional mechanisms, including translational control and enzyme kinetics and stability, to explain the disconnect they observed between the gene transcript ratio (*cyr*J:*cyr*I) and the toxin ratio (deoxyCYN:CYN).

It is important to consider the changes observed in this study in the toxin profiles of the strains, particularly the increase of more toxic analogs under nutrient‐reduced conditions. This increase indicates that although a reduction of nutrient levels in the aquatic systems could have a positive outcome for the control of cyanobacterial blooms (Carpenter, 2008; Giani et al., 2020; Schindler et al., 2016), it could have a negative effect by increasing bloom toxicity under nutrient‐reduced conditions. This makes expanding our understanding of how toxin production and composition are modulated by nutrient supply in different cyanobacterial species critical (Hellweger et al., 2022). Moving forward nutrient‐dependent gene regulation, such as the patterns observed here, may be an important tool for screening field populations and predicting how resources may be shaping the overall toxicity of cyanobacterial populations.

## AUTHOR CONTRIBUTIONS

**Mehrzad Zare:** Data curation (lead); formal analysis (lead); investigation (lead); methodology (equal); validation (equal); visualization (equal); writing – original draft (lead); writing – review and editing (equal). **Bruna Barçante:** Data curation (supporting); formal analysis (equal); methodology (equal); validation (equal); visualization (supporting); writing – review and editing (supporting). **Juliana da Silva Martins Pimentel:** Formal analysis (supporting); methodology (equal); supervision (supporting); validation (equal); writing – review and editing (supporting). **Alessandra Giani:** Conceptualization (lead); data curation (supporting); funding acquisition (lead); investigation (supporting); project administration (lead); resources (lead); supervision (lead); validation (equal); visualization (equal); writing – review and editing (equal).

## Supporting information


**Figure S1.** Mean values of cell numbers in each experiment performed in replicate.

**Figure S2.** HPLC‐FD chromatogram of the mixed saxitoxin standard solution. The peaks represent: 1 to 4‐ gonyautoxins (GTX4, GTX1, GTX3, GTX2), 5‐ neosaxitoxin (NeoSTX), 6‐ decarbamoyl‐saxitoxin (dcSTX) and 7‐ saxitoxin (STX), respectively.
**Figure S3.** HPLC‐FD chromatograms of STX analogs in *R. raciborskii* UFMG‐36 in nitrogen growth experiments. The peaks represent: 1‐ GTX4; 2‐ GTX1; 3‐ GTX3; 4‐ GTX2, 5‐ NeoSTX; 6‐ dcSTX; 7‐ STX, respectively.
**Figure S4.** HPLC‐FD chromatograms of STX analogs in *R. raciborskii* UFMG‐36 in phosphorus growth experiments. The peaks represent: 2‐ GTX1; 3‐ GTX3; 4‐ GTX26‐ dcSTX, respectively.
**Figure S5.** HPLC‐FD chromatograms of STX analogs in *R. raciborskii* UFMG‐186 in nitrogen growth experiments. The peaks represent: 3‐ GTX3; 4‐ GTX2, respectively.
**Figure S6.** HPLC‐FD chromatograms of STX analogs in *R. raciborskii* UFMG‐186 in phosphorus growth experiments. The peaks represent: 1‐ GTX4; 3‐ GTX3; 4‐ GTX2, 7‐ STX, respectively.

## Data Availability

All data from these experiments will be made available upon contacting the corresponding author.
