# Supplementary material for: Nutrient‐driven regulation of saxitoxin gene expression and toxin production in Raphidiopsis raciborskii (Cyanobacteria)
Source: J Phycol. 2025 Dec 3;61(6):1738–52. doi: 10.1111/jpy.70115 (PMC12718436; doi:10.1111/jpy.70115)
Supplement: Supplementary file 1 — Figure S1. Mean values of cell numbers in each experiment performed in replicate. [file JPY-61-1738-s001.docx]

**Figure S1**

Figure S1. Mean values of cell numbers in each experiment performed in replicate. Different letters above the columns indicate statistical difference among treatments (*p* ≤ 0.05). Whiskers represent standard errors (not visible when *SE* very small). Tables below each figure show basic statistics.
