# Supplementary material for: Nutrient‐driven regulation of saxitoxin gene expression and toxin production in Raphidiopsis raciborskii (Cyanobacteria)
Source: J Phycol. 2025 Dec 3;61(6):1738–52. doi: 10.1111/jpy.70115 (PMC12718436; doi:10.1111/jpy.70115)
Supplement: Supplementary file 2 — Figure S2. HPLC‐FD chromatogram of the mixed saxitoxin standard solution. The peaks represent: 1 to 4‐ gonyautoxins (GTX4, GTX1, GTX3, GTX2), 5‐ neosaxitoxin (NeoSTX), 6‐ decarbamoyl‐saxitoxin (dcSTX) and 7‐ saxitoxin (STX), respectively. Figure S3. HPLC‐FD chromatograms of STX analogs in R. raciborskii UFMG‐36 in nitrogen growth experiments. The peaks represent: 1‐ GTX4; 2‐ GTX1; 3‐ GTX3; 4‐ GTX2, 5‐ NeoSTX; 6‐ dcSTX; 7‐ STX, respectively. Figure S4. HPLC‐FD chromatograms of STX analogs in R. raciborskii UFMG‐36 in phosphorus growth experiments. The peaks represent: 2‐ GTX1; 3‐ GTX3; 4‐ GTX26‐ dcSTX, respectively. Figure S5. HPLC‐FD chromatograms of STX analogs in R. raciborskii UFMG‐186 in nitrogen growth experiments. The peaks represent: 3‐ GTX3; 4‐ GTX2, respectively. Figure S6. HPLC‐FD chromatograms of STX analogs in R. raciborskii UFMG‐186 in phosphorus growth experiments. The peaks represent: 1‐ GTX4; 3‐ GTX3; 4‐ GTX2, 7‐ STX, respectively. [file JPY-61-1738-s002.docx]

**Figure S2–S6**

Figure S2. HPLC-FD chromatogram of the mixed saxitoxin standard solution. The peaks represent:

1 to 4- gonyautoxins (GTX4, GTX1, GTX3, GTX2), 5- neosaxitoxin (NeoSTX), 6- decarbamoyl-saxitoxin (dcSTX) and 7- saxitoxin (STX), respectively.

36- 1% N

36- 10% N

36- 100% N (control)

Figure S3. HPLC-FD chromatograms of STX analogs in *R. raciborskii* UFMG-36 in nitrogen growth experiments. The peaks represent: 1- GTX4; 2- GTX1; 3- GTX3; 4- GTX2, 5- NeoSTX; 6- dcSTX; 7- STX, respectively.

36- 1% P

36- 10% P

36- 100% P (control)

Figure S4. HPLC-FD chromatograms of STX analogs in *R. raciborskii* UFMG-36 in phosphorus growth experiments. The peaks represent: 2- GTX1; 3- GTX3; 4- GTX26- dcSTX, respectively.

186- 1% N

186- 10% N

186- 100% N (control)

Figure S5. HPLC-FD chromatograms of STX analogs in *R. raciborskii* UFMG-186 in nitrogen growth experiments. The peaks represent: 3- GTX3; 4- GTX2, respectively

186- 1% P

186- 10% P

186- 100% P (control)

Figure S6. HPLC-FD chromatograms of STX analogs in *R. raciborskii* UFMG-186 in phosphorus growth experiments. The peaks represent: 1- GTX4; 3- GTX3; 4- GTX2, 7- STX, respectively.
